# Supplementary material for: Interaction of human keratinocytes and nerve fiber terminals at the neuro-cutaneous unit
Source: eLife. 2024 Jan 16;13:e77761. doi: 10.7554/eLife.77761 (PMC10791129; doi:10.7554/eLife.77761)
Supplement: Transparent reporting form [file elife-77761-transrepform1.docx]

***eLife’s* transparent reporting form**

We encourage authors to provide detailed information *within their submission* to facilitate the interpretation and replication of experiments. Authors can upload supporting documentation to indicate the use of appropriate reporting guidelines for health-related research (see [EQUATOR Network](http://www.equator-network.org/%20)), life science research (see the [BioSharing Information Resource](https://biosharing.org/)), or the [ARRIVE guidelines](http://www.plosbiology.org/article/info:doi/10.1371/journal.pbio.1000412) for reporting work involving animal research. Where applicable, authors should refer to any relevant reporting standards documents in this form.

If you have any questions, please consult our Journal Policies and/or contact us: [editorial@elifesciences.org](mailto:editorial@elifesciences.org).

**Sample-size estimation**

- You should state whether an appropriate sample size was computed when the study was being designed
- You should state the statistical method of sample size computation and any required assumptions
- If no explicit power analysis was used, you should describe how you decided what sample (replicate) size (number) to use

Please outline where this information can be found within the submission (e.g., sections or figure legends), or explain why this information doesn’t apply to your submission:

No power analysis was performed.

Due to the descriptive nature of our imaging approached, which included extensive sample preparation, imaging, and post-imaging analysis we do not include statistical analysis of the observed interactions. We aimed to validate our results via different imaging approaches (srAT, ExM) and conditions (*ex vivo* tissue, *in vitro* model).

**Replicates**

- You should report how often each experiment was performed
- You should include a definition of biological versus technical replication
- The data obtained should be provided and sufficient information should be provided to indicate the number of independent biological and/or technical replicates
- If you encountered any outliers, you should describe how these were handled
- Criteria for exclusion/inclusion of data should be clearly stated
- High-throughput sequence data should be uploaded before submission, with a private link for reviewers provided (these are available from both GEO and ArrayExpress)

Please outline where this information can be found within the submission (e.g., sections or figure legends), or explain why this information doesn’t apply to your submission:

SrAT was applied to tissue samples derived from n=3 (one female and two males) due to extensive sample preparation and image analysis.

Initial ExM (Figure 3 & 5) was applied to at least 2 clinical sections each from 3 healthy controls due to extensive sample preparation and image analysis. Quantitative assessment of ExM ROIs was performed with 5 healthy control samples (n = 13 ROIs) versus 4 SFN samples (n = 10 ROIs).

For the *in vitro* model 6 rounds of co-culturing with each several coverslips were performed for various target labeling. 6 neuronal differentiations from one clone of 1 healthy iPSC line and 1 keratinocyte line from a healthy control were used to minimize variability. For labeling panels of Cx43, Ctx, and WGA (Figure 7) two coverslips were used, while for panels of Ctx, SYP,WGA (Figure 8) and Ctx, panNF, SYP (Figure 8—figure supplement 1) each one coverslips was used.

For Ca2+ imaging one neuronal differentiation and one keratinocyte line was used with 7 wells as technical replicates.

**Statistical reporting**

- Statistical analysis methods should be described and justified
- Raw data should be presented in figures whenever informative to do so (typically when N per group is less than 10)
- For each experiment, you should identify the statistical tests used, exact values of N, definitions of center, methods of multiple test correction, and dispersion and precision measures (e.g., mean, median, SD, SEM, confidence intervals; and, for the major substantive results, a measure of effect size (e.g., Pearson's r, Cohen's d)
- Report exact p-values wherever possible alongside the summary statistics and 95% confidence intervals. These should be reported for all key questions and not only when the p-value is less than 0.05.

Please outline where this information can be found within the submission (e.g., sections or figure legends), or explain why this information doesn’t apply to your submission:

Information on statistical methods are reported at methods section. Input data and statistical details (median; SD; SEM, etc.) for Figure 5 are summarized in Figure 5-source data 1 and Figure 5-source data 2.

(For large datasets, or papers with a very large number of statistical tests, you may upload a single table file with tests, Ns, etc., with reference to sections in the manuscript.)

**Group allocation**

- Indicate how samples were allocated into experimental groups (in the case of clinical studies, please specify allocation to treatment method); if randomization was used, please also state if restricted randomization was applied
- Indicate if masking was used during group allocation, data collection and/or data analysis

Please outline where this information can be found within the submission (e.g., sections or figure legends), or explain why this information doesn’t apply to your submission:

Experimental groups: not applicable, since this is not a clinical study.

Masking: data was analyzed in a blinded manner as described in the Methods section.

**Additional data files (“source data”)**

- We encourage you to upload relevant additional data files, such as numerical data that are represented as a graph in a figure, or as a summary table
- Where provided, these should be in the most useful format, and they can be uploaded as “Source data” files linked to a main figure or table
- Include model definition files including the full list of parameters used
- Include code used for data analysis (e.g., R, MatLab)
- Avoid stating that data files are “available upon request”

Please indicate the figures or tables for which source data files have been provided:

We provide source data files for the 3D reconstruction of tracked intraepidermal fiber via srAT (Figure 1). The model file, fluorescent signal stacks and electron microscopy image stacks are available at zenodo.org under the following link:

<https://zenodo.org/record/6090262#.YgykxRJOeUk>

Further supplementary file1 (excel sheet) contains information about used samples and participants, Supplementary file 2 (zip folder) contains applied FIJI and CellProfiler pipelines, Figure 5-source data 1 contains nuclear expansion size calculations from Figure 5, and Figure 5-source data 2 contains input values and statistical analysis of Figure 5 graphs between HC and SFN. Further we provide 6 Videos for z-stack and timelapse data and are referred to at their corresponding figure.
